# RETRACTION: Luteolin Pretreatment Ameliorates Myocardial Ischemia/Reperfusion Injury by lncRNA‐JPX/miR‐146b Axis

**DOI:** 10.1155/ancp/9805734

**Published:** 2026-06-10

**Authors:** Analytical Cellular Pathology

RETRACTION: T. Xu, Y. Zhang, G. Liao,et al., “Luteolin Pretreatment Ameliorates Myocardial Ischemia/Reperfusion Injury by lncRNA‐JPX/miR‐146b Axis,” *Analytical Cellular Pathology*, 2023, 4500810, https://doi.org/10.1155/2023/4500810.

The above article, published online on 2 December 2023 in Wiley Online Library (wileyonlinelibrary.com), has been retracted by agreement between the journal Editor‐in‐Chief, Dimitrios Karamichos; and John Wiley & Sons Ltd. The retraction has been agreed due to concerns identified with the Figure 4, some of which were reported on PubPeer [1]. Further investigation by the Publisher found additional issues with Figure 4. A summary of all concerns is as follows:

• There are unexpected, repeated regions between the Lut and the Ad‐JPX+Lut panels shown in Figure 4f
• There are highly similar regions between the Con and Lut micrographs shown in Figure 4e
• There are highly similar regions between the Ad‐EGFP and Ad‐JPX+Lut micrographs shown in Figure 4e

Additionally, concerns were raised regarding the apoptosis measurements in Figure 4e.

After assessment of the concerns and author’s response by the editorial board, the results and conclusions can no longer be considered reliable, and the article is retracted.

The authors disagree with the retraction.
